# Sociodemographic and Health Correlates of Multiple Health Behavior Adherence among Cancer Survivors: A Latent Class Analysis

**DOI:** 10.3390/nu15102354

**Published:** 2023-05-17

**Authors:** Angela J. Fong, Adana A. M. Llanos, Adiba Ashrafi, Nur Zeinomar, Sagar Chokshi, Elisa V. Bandera, Katie A. Devine, Shawna V. Hudson, Bo Qin, Denalee O’Malley, Lisa E. Paddock, Antoinette M. Stroup, Andrew M. Evens, Sharon L. Manne

**Affiliations:** 1 Section of Behavioral Sciences, Rutgers Cancer Institute of New Jersey, New Brunswick, NJ 08901, USA; 2 Division of Medical Oncology, Department of Medicine, Rutgers Robert Wood Johnson Medical School, New Brunswick, NJ 08901, USA; 3 Department of Epidemiology, Mailman School of Public Health, Columbia University Irving Medical Center, New York, NY 10032, USA; 4 Herbert Irving Comprehensive Cancer Center, Columbia University Irving Medical Center, New York, NY 10032, USA; 5 Section of Cancer Epidemiology and Health Outcomes, Rutgers Cancer Institute of New Jersey, New Brunswick, NJ 08901, USA; 6 Department of Obstetrics, Gynecology, and Reproductive Sciences, Rutgers Robert Wood Johnson University Hospital, New Brunswick, NJ 08901, USA; 7 Department of Pediatrics, Section of Pediatric Population Science, Rutgers Cancer Institute of New Jersey, New Brunswick, NJ 08901, USA; 8 Department of Family Medicine and Community Health, Rutgers Robert Wood Johnson Medical School, New Brunswick, NJ 08901, USA; 9 Rutgers Cancer Institute of New Jersey, New Brunswick, NJ 08901, USA; 10 Department of Biostatistics and Epidemiology, Rutgers School of Public Health, Piscataway, NJ 08854, USA; 11 Department of Medicine, Division of Blood Disorders, Rutgers Robert Wood Johnson Medical School, New Brunswick, NJ 08901, USA

**Keywords:** cancer survivors, survivorship, multiple health behaviors, correlates, sociodemographic, latent class analysis

## Abstract

The study aimed to (a) assess current levels of adherence to the National Comprehensive Cancer Network’s multiple health behavior guidelines and (b) identify characteristics of cancer survivors associated with different adherence levels. Cancer survivors (*N* = 661) were identified through the state registry and completed questionnaires. Latent class analysis (LCA) was used to identify patterns of adherence. Associations between predictors with the latent classes were reported as risk ratios. LCA identified three classes: lower- (39.6%), moderate- (52.0%), and high-risk lifestyle (8.3%). Participants in the lower-risk lifestyle class had the highest probability of meeting most of the multiple health behavior guidelines compared to participants in the high-risk lifestyle class. Characteristics associated with membership in the moderate-risk lifestyle class included self-identifying as a race other than Asian/Asian American, being never married, having some college education, and having been diagnosed with later stage colorectal or lung cancer. Those in the high-risk lifestyle class were more likely to be male, never married, have a high school diploma or less, diagnosed with colorectal or lung cancer, and diagnosed with pulmonary comorbidities. Study findings can be used to inform development of future interventions to promote multiple health behavior adherence among higher risk cancer survivors.

## 1. Introduction

There are 18.1 million Americans alive today with a history of cancer, and this number is expected to reach 26 million by 2040 [1,2]. As cancer survivors live longer, they are expected to experience cancer-related long-term physical and psychological sequalae [3]. They are at an increased risk of developing cardiovascular disease, diabetes, dyslipidemia, osteoporosis, and functional decline compared to individuals without a history of cancer diagnosis [4,5,6]. Additionally, cancer survivors die of preventable non-cancer causes, such as cardiovascular disease, at a higher rate than persons in the general population [7]. This is likely due, in part, that many cancers share risk factors such as obesity, physical inactivity, smoking with cardiovascular disease. Thus, it is imperative to develop strategies which manage cardio-metabolic comorbidities and mitigate risk factors of all-cause mortality among cancer survivors.

Engaging in multiple health behaviors—such as being physically activity, eating a healthy dietary pattern, restricting alcohol consumption, and not smoking—reduce cancer-related morbidity and all-cause mortality among cancer survivors [8,9]. Furthermore, convincing data suggest that obesity is associated with increased mortality in most malignancies and maintaining a healthy body mass index (BMI) is important [10,11,12]. It is also common to cluster multiple health behaviors together, such as increasing physical activity and eating a healthy diet [13]. In an effort to reduce risk of cancer recurrence and death, and promote a healthy lifestyle among cancer survivors, the National Comprehensive Cancer Network (NCCN) has included healthy lifestyle recommendations in their survivorship guidelines [14]. These recommendations include eight goals: (1) engaging in physical activity/avoiding inactivity (i.e., 150 min of moderate-to-vigorous physical activity per week), (2) achieving and maintaining a healthy body weight (i.e., BMI = 18.5–24.9 kg/m^2^), (3) maintaining a healthy diet high in vegetables, fruits, and whole grains, (4) abstaining or limiting alcohol consumption, (5) avoiding tobacco products, (6) practicing sun safety, (7) getting enough sleep, and (8) seeking regular follow-up care with a primary care provider (i.e., every 6 months or more) [14]. Meeting guidelines for physical activity, body weight, smoking, alcohol consumption, and sun safety has numerous health benefits for a high-risk population such as cancer survivors [15]. For example, a recent study reported reduced risk of cancer-specific and all-cause mortality for breast cancer survivors with a healthy lifestyle, based on a composite measurement of modifiable risk factors, such as BMI, physical activity, intake of plant and animal foods, alcohol consumption, and smoking [16]. While a cancer diagnosis presents an opportunity to promote and foster better health behaviors, studies show that many survivors do not adhere to multiple lifestyle recommendations and identifying factors that contribute to adherence is needed [17,18,19,20,21].

Recent research suggests that sociodemographic, cancer-, and health-related factors are associated with adherence to both single- and multiple health behavior recommendations among cancer survivors [22,23]. Cancer survivors who were biologically male, non-Hispanic White or Black, with a high school education or less, not married, and diagnosed with comorbidities were more likely to consume alcohol, smoke cigarettes or e-cigarettes, and have a physically inactive lifestyle [22]. Similarly, racial and ethnic differences have been characterized for alcohol consumption (e.g., non-Hispanic Whites more commonly reported) and higher BMI (e.g., Black individuals more commonly reported) among breast cancer survivors [24]. While studies have characterized differences in health behaviors by race and ethnicity, it is unclear which characteristics (i.e., sociodemographic, socioeconomic status, cancer-related factors, and comorbidities) of cancer survivors are associated with lower adherence to NCCN guidelines for multiple health behaviors. Since health behaviors are modifiable, identifying the subpopulations of cancer survivors who are least likely to adhere to recommendations would allow for interventions to be tailored for the most at-risk cancer survivors [23]. The study aimed to (a) assess current levels of adherence to NCCN guidelines for multiple health behaviors among cancer survivors and (b) identify and compare subgroups/characteristics of cancer survivors associated with the different levels of adherence.

## 2. Materials and Methods

### 2.1. Participants

Participants were identified through the New Jersey State Cancer Registry (NJSCR) and eligibility was confirmed by the diagnosing physician and research staff. Eligibility criteria included: (a) 18–85 years of age; (b) a current resident of New Jersey; (c) diagnosis in 2015 or 2016 with a primary case of genitourinary (i.e., bladder and prostate), female breast, gynecologic (i.e., cervical, endometrial, ovarian), colorectal, lung, melanoma, or thyroid cancer; and (d) ability to read and speak English. This study received institutional review board approval at the host institution.

### 2.2. Procedures

In this cross-sectional study, eligible cancer survivors were sent a recruitment package that included a cover letter, study information, questionnaire, and postage-paid return envelope between August 2018 to January 2022. One week after the mailing, NJSCR staff called potential participants by telephone to confirm receipt of the recruitment package and answer any questions participants had prior to returning their questionnaires. Participants received up to eight follow-up phone calls on weekdays and weekends to remind them to complete and return the questionnaire. Individuals who returned the questionnaire were given a $25 gift card in appreciation for their time and participation. A total of 3012 individuals were contacted and among them, 29 were deceased, 175 were deemed ineligible, 116 were categorized as lost, 1830 refused (484 active and 1346 passive), and 862 returned the questionnaire (28.6% response rate). Of the 862 respondents, 201 had missing data for at least one of the seven outcomes of interest, and were therefore excluded, leaving 661 cancer survivors in the analytic sample. Compared to responders, the non-responders did have a significantly higher proportion of late-stage cancer survivors (29.4% vs. 20.7%, *p* = 0.037) and those whose household income was unknown (28.9% vs. 19.2%, *p* = 0.027) (Supplementary Table S1). More notably, the 1830 non-responders comprised of a significantly higher proportion of cancer survivors who self-identified as Black/African American (18.0% vs. 11.3%) and Asian/Asian American (8.6% vs. 5.9%) when compared to responders that were included in the analytic sample (*p* < 0.001). The non-responders also had a significantly lower proportion of breast cancer survivors (19.1% vs. 25.6%), but a higher proportion of gynecologic cancer survivors (13.1% vs. 10.1%) when compared to responders (*p* = 0.004). There were no other significant differences in sociodemographic characteristics (*p* > 0.05) between responders and non-responders.

### 2.3. Measures

#### 2.3.1. Subpopulation Characteristics

Sociodemographic. All available data for self-identified sex (male or female), race (White, Black or African American, Asian or Asian American, or other [including American Indian or Alaska Native, Native Hawaiian or other Pacific Islander, and multiracial]), and Hispanic ethnicity (yes or no) were included, and any values missing from the questionnaire were imputed with NJSCR registry data. Age (in years) at cancer diagnosis was calculated as the difference between the patient’s birthdate and date of cancer diagnosis. Other self-reported sociodemographic variables included nativity (US-born [yes or no]) and marital status (married [including separated couples], unmarried [divorced or widowed], or single/never married).

Socioeconomic Status (SES). Self-reported SES data included education (high school graduate or less, some college/post high school, college graduate, or post college [graduate degree, professional degree, or other certifications/credits following a college degree]), employment status (employed/self-employed, unemployed/homemaker/student/disabled, or retired), annual household income (<$50,000, $50,000–$89,999, and ≥$90,000), and health insurance status (uninsured, private, public [Medicaid or Medicare], or insured—not otherwise specified).

Cancer-related Factors. Participants also self-reported their primary cancer diagnosis (female breast, colorectal, genitourinary [urinary bladder or prostate], gynecologic [vulvar, endocervical, cervical, endometrial, myometrium, corpus uteri, and ovarian], lung, malignant skin [melanoma], or thyroid). Cancer stage was determined based on the year of diagnosis and staging mechanism; participants were classified as unstaged, early stage (in situ and localized cases), or late stage (regional and distant cases). Time (in years) since cancer diagnosis was calculated as the difference between the date of cancer diagnosis and date of survey completion. Receipt of cancer treatment (surgery, chemotherapy, or radiation therapy) was recorded (yes, no, or do not recall) and through a count of how many modalities were received (0, 1, or ≥2).

Comorbidities. A checklist of 23 health conditions derived from the Health Information National Trends Survey (HINTS) [25] was included in the questionnaire to assess participants’ history of cardiovascular and metabolic (0, 1, or ≥2), pulmonary (0 or ≥1), and other (0 or ≥1) comorbidities. Cardiovascular and metabolic comorbidities included a history of diabetes, hypertension, hypercholesterolemia, heart disease, angina, heart attack, congestive heart failure, myocardial infarction, kidney disease, and/or liver disease; pulmonary comorbidities included a history of emphysema/chronic obstructive pulmonary disease (COPD), and/or asthma; and other comorbidities included a history of depression, anxiety disorder, schizophrenia, bipolar disorder, post-traumatic stress disorder (PTSD), peripheral vascular disease. cerebrovascular disease, dementia, connective tissue disease, leukemia, malignant lymphoma, hematological or solid tumor, and/or acquired immunodeficiency syndrome (AIDS).

#### 2.3.2. Current Health Behaviors

Body Mass Index (BMI). BMI (in kg/m^2^) at the time of questionnaire completion was computed using participants’ participants’ reported height (feet) and weight (pounds) and converted to metric (i.e., meters and kilograms).

Physical Activity. Physical activity was measured using the Godin Leisure Time Exercise Questionnaire [26]. The 4-item measure asks participants to report the number of sessions over the previous 7 days when they engage in 30 min or more of mild (e.g., light walking), moderate (e.g., walking at a brisk pace), and strenuous (e.g., running) activity and how often they sweat during a session (i.e., sometimes, often, never). A score is calculated by multiplying the number of mild sessions by three, the number of moderate sessions by five, and the number of strenuous sessions by nine. Participants with a Leisure Score Index (LSI) of <14 were classified as insufficiently active/sedentary, those with scores ranging from 14 to 23 were catalogued as moderately active, and those with scores of ≥24 were considered active participants fulfilling NCCN standards. This measure has demonstrated acceptable reliability and validity among cancer survivor populations [27].

Alcohol Consumption. Two items from the FOCUS questionnaire [28] were used to assess alcohol consumption levels (0, 0 to ≤1, 1 to 2, or >2 drinks/day). The items were “Have you had any beer, wine, wine coolers, mixed drinks, liquor or other alcoholic beverages during the past month?” (yes/no response) and “During the past month, on the days you drank alcoholic beverages, about how many drinks did you drink on average?” (response in number of drinks) informed how many drinks per day participants consumed, if any.

Smoking Status. Three items from the Follow-up Care Use and Health Outcomes Survey (FOCUS) questionnaire [28] were used to assess smoking status (never smoker, former smoker, or current smoker). The items were “Have you smoked at least 100 cigarettes in your entire lifetime?” distinguished ever (yes response) vs. never smokers (no response), and the questions “How often do you smoke cigarettes now?” (responses included every day, some days, and not at all) and “How many days in the past 30 days did you smoke?” (response in number of days) separated former vs. current smokers.

Healthy Diet Intake. A 7-item measure was developed for this study. The cancer-specific dietary recommendations from the World Cancer Research Fund/American Institute of Cancer Research were used to assess food frequency over the past week [29]. Food categories included fruits, vegetables, sugar-sweetened beverages, whole grain products, red meat, processed meat, dairy products, and fast foods. Responses were rated on a 6-point scale: 1 = “never”, 2 = ”once/week”, 3 = ”2–4 times/week”, 4 = ”5–6 times/week”, 5 = ”once/day”, and 6 = ”≥2 times/day”. Items for sugar-sweetened beverages, red meat, processed meat, and fast foods were reverse coded. Intake of an overall healthy diet was then ascertained by computing the mean across all items of all food categories, where a higher score indicates closer alignment with the nutritional recommendations [30].

Sun Safety. Five items from the HINTS were used to assess sun safety [25]. The questionnaire items were: (a) “How often do you wear sunscreen?”; (b) “How often do you wear a shirt with sleeves that cover your shoulders?”; (c) “How often do you wear a hat?”; (d) “How often do you stay in the shade or under an umbrella?”; and (e) “How often do you wear sunglasses?”. Responses were collected using a 5-point Likert scale, where 1 = “never”, 2 = “rarely”, 3 = “sometimes”, 4 = “often”, and 5 = “always.”

Regular Follow-Ups with A Primary Care Provider. A single item derived from the Medical Expenditure Panel Survey [31] was used to assess when participants last visited their physician or healthcare provider. The item is “When did you last see any doctor for your cancer-related care?” and it was rated as <4 weeks ago, 1–3 months ago, 4–6 months ago, 7–12 months ago, and >2 years ago.

### 2.4. Data Analysis

Descriptive statistics were used to characterize sociodemographic and health history characteristics of participants in the analytic sample. To identify distinct patterns of health behaviors among cohort members, latent class analysis (LCA) was performed, using both categorical (BMI, physical activity, smoking status, alcohol consumption, and last physician visit) and continuous (healthy diet intake and sun safety) health behavior items. A series of LCA models between two and six classes were tested to determine the number of classes that best represented mutually exclusive health behavior patterns among cancer survivors in this cohort (Supplementary Table S2). Models were run using randomly generated seed values.

The Bayesian Information Criterion (BIC) was generated for each latent class model, where a lower BIC indicates better goodness of fit. To compare models, the Vuong–Lo–Mendell–Rubin likelihood ratio test (LRT) was also examined; a significant *p*-value indicates that the estimated model fits the data better than a model with one fewer class. To determine the appropriate number of latent classes, we evaluated the interpretability of each model and compared their BIC and LRT *p*-values.

After a latent class model was chosen, each cancer survivor was assigned to the class for which they had the highest probability of membership. Differences in health behaviors of cancer survivors across latent classes were assessed with pairwise comparisons. Specifically, pairwise comparisons of proportions across health behavior classes were evaluated using two-sided Chi-Squared tests (assuming equal variances), and pairwise comparisons of means were assessed using analysis of variance (ANOVA). The Benjamini–Hochberg (BH) approach was used to correct for multiple comparisons, and each significant pair following correction at a significance level of 5% was identified.

Subsequently, relationships between sociodemographic and health history factors and latent health behavior classes were examined using latent class regression—more specifically, by running a generalized logit model with a multinomial distribution, and robust error variances. The sociodemographic and health history factors of interest were sex, age at cancer diagnosis, race, marital status, education, employment status, primary cancer diagnosis, cancer stage at diagnosis, number of cancer treatment modalities received, and number of cardiometabolic and pulmonary comorbidities reported. For each categorical variable, missing responses or individuals who responded “other”, “don’t know”, or “prefer not to answer” were combined into a separate level of that covariate so that observations would not be excluded from the LCA regression model due to missing or non-specific responses. Continuous covariates did not have any missing observations. Associations were reported as odds ratios (ORs) and significant *p*-values were identified using the BH procedure. SPSS (version 26.0), Mplus (version 8.8), STATA (version 16.1), and GraphPad Prism (version 9.4.1) were used to compute descriptive statistics, conduct latent class analysis, perform latent class regression analyses, and illustrate log ORs, respectively.

## 3. Results

### 3.1. Participant Characteristics

Table 1 presents participant characteristics. Participants (*N* = 661) were predominantly female (58.7%), an average of 60.8 (*SD* = 10.5) years old when diagnosed with cancer, and mostly self-identified as White (78.4%) and non-Hispanic (94.1%). Furthermore, most participants were born in the United States (86.1%), married (68.4%), college graduates (30.4%), and either employed/self-employed (43.0%) or retired (41.9%) at the time of survey completion. Approximately one-quarter reported having a household income <$50,000 (24.4%).

The primary cancer diagnoses were heterogeneous, with genitourinary (27.7%) and breast (25.6%) cancers being the most prevalent. More than three-quarters of participants (77.9%) relayed that their primary cancer was at an early stage when diagnosed. Time since cancer diagnosis ranged from 2 to 5 years, with an average of 3.2 (*SD* = 0.7) years. Participants received surgery (82.5%), chemotherapy (27.1%), and radiotherapy (39.0%) as treatments for their cancers. Very few had not received any cancer treatment (4.1%) at the time of survey completion and even fewer were uninsured (1.2%). Having ≥2 cardiometabolic comorbidities was commonly reported (43.1%).

A large majority of participants were either overweight or obese, as their BMIs fell within the range of 25.0–29.9 kg/m^2^ (36.3%) or ≥30.0 kg/m^2^ (34.3%) (Table 2). Nearly half of the participants (48.1%) stated that they were active (LSI ≥ 24), nearly one-third (32.4%) were sedentary (LSI < 14). Most participants were never smokers (51.4%) and consumed zero drinks of alcohol per day (36.2%). In addition, the one-third had seen a physician in the last 1–3 months (30.9%). In terms of diet, participants commonly reported eating fruits and whole grains 2–4 times/week (26.6% and 33.4%, respectively) and vegetables once/day (26.2%). They also frequently described never consuming sugar (65.4%), eating red meat 2–4 times a week (44.0%), and eating processed meat and fast foods once/week (23.9% and 57.8%, respectively). With regards to sun safety, participants recounted ‘always’ wearing shirts with sleeves (36.8%) and sunglasses (44.8%), ‘often’ staying in the shade or under an umbrella (37.4%), and only ‘sometimes’ wearing sunscreen (23.1%) or a hat (26.8%).

Altogether, most cancer survivors were non-adherent to NCCN guidelines for maintaining a healthy weight (71.4%) and staying active (51.9%) (Table 3). Participants were non-adherent to NCCN guidelines for smoking (23.8%) and sun safety (29.7%). In contrast, the majority were adherent to NCCN guidelines for a healthy diet intake (97.9%) and seeking follow-up care by attending physician visits (at least within the past 12 months) (93.3%).

### 3.2. Differences in Health Behaviors by Latent Class Membership

LCA identified a 3-class model as the best fit and most interpretable model for describing distinct health behavior patterns among cancer survivors in this cohort (Supplementary Table S2). Item response probabilities and means for health behavior items in all three latent classes are shown in Supplementary Table S3. Participants were divided into the three latent classes based on their most likely class membership. Class membership was characterized by health behavior patterns: (A) high-risk lifestyle (*N* = 55, 8.3%), (B) moderate-risk lifestyle (*N* = 344, 52.0%), and (C) lower-risk lifestyle (*N* = 262, 39.6%) (Table 2).

### 3.3. Lower-Risk Lifestyle

Participants with a lower-risk lifestyle followed the NCCN guidelines to all health behaviors, except alcohol consumption, more frequently than participants in at least one, and often both other classes (Table 2 and Table 3). Compared to the high- and moderate-risk lifestyle classes, the lower-risk lifestyle class had a significantly higher proportion of participants with a healthy weight (35.1% vs. 18.2% and 25.3%, *p* = 0.022), who were physically active (63.7% vs. 32.7% and 38.7%, *p* < 0.001) and never smokers (59.2% vs. 30.9% and 48.8%, *p* < 0.05). Generally, participants in this class followed the nutritional recommendations of the World Cancer Research Fund/American Institute of Cancer Research more than participants in the high-risk and moderate-risk lifestyle classes. That is, the mean for each healthy diet intake item was significantly higher than that of the other two classes (*p* < 0.001), indicating that, on average, they consumed more fruits (mean ± *SD*: 5.1 ± 0.9 vs. 3.3 ± 1.5 and 3.0 ± 1.1), vegetables (mean ± *SD*: 5.4 ± 0.6 vs. 3.7 ± 1.4 and 3.5 ± 1.0), and whole grains (mean[*SD*]: 4.2 ± 1.4 vs. 3.6 ± 1.5 and 2.8 ± 1.1), as well as less sugar (mean ± *SD*: 5.8 ± 0.5 vs. 2.2 ± 0.8 and 5.5 ± 0.7), red meat (mean ± *SD*: 4.6 ± 1.0 vs. 3.7 ± 1.3 and 4.3 ± 0.8), processed meat (mean ± *SD*: 5.1 ± 0.9 vs. 4.1 ± 1.2 and 4.6 + 0.9), and fast foods (mean ± *SD*: 5.3 ± 0.6 vs. 4.5 ± 0.9 and 4.9 ± 0.8) than their counterparts. On average, participants with a lower-risk lifestyle applied more sunscreen (mean ± *SD*: 3.4 ± 1.3 vs. 2.6 ± 1.5 and 2.8 ± 1.4, *p* < 0.001) and wore sunglasses more frequently (mean ± *SD*: 4.2 ± 1.1 vs. 3.7 ± 1.4, *p* < 0.05) than those in the other two classes. Lower-risk participants also wore hats more often (mean ± *SD*: 3.2 ± 1.3 vs. 2.8 ± 1.3, *p* = 0.006) when compared to moderate-risk lifestyle participants. Those with a lower-risk lifestyle visited their physician more regularly in the past year (95.8% vs. 87.3%, *p* = 0.039) compared to high-risk lifestyle participants.

### 3.4. Moderate-Risk Lifestyle

Participants with a moderate-risk lifestyle did not adhere to as many NCCN guidelines for multiple health behaviors as those in the lower-risk lifestyle class, but they did adhere to more than those with a high-risk lifestyle (Table 2 and Table 3). On average, participants with a moderate-risk lifestyle consumed significantly less sugar (mean ± *SD*: 5.5 ± 0.7 vs. 2.2 ± 0.8, *p* < 0.001), red meat (mean ± *SD*: 4.3 ± 0.8 vs. 3.7 ± 1.3, *p* < 0.001), processed meat (mean ± *SD*: 4.6 ± 0.9 vs. 4.1 ± 1.2, *p* < 0.001), and fast foods (mean ± *SD*: 4.9 ± 0.8 vs. 4.5 ± 0.9, *p* < 0.001) than participants with a high-risk lifestyle. In terms of healthy diet intake overall (mean score >3 for all 7 food items), a higher proportion of moderate- versus high-risk lifestyle participants were adherent to the NCCN guidelines (98.8% vs. 81.8%, *p* < 0.001). The moderate-risk lifestyle class also had a significantly higher percentage of never smokers than the high-risk lifestyle class (48.8% vs. 30.9%, *p* = 0.013), as well as a significantly higher proportion of current smokers than the lower-risk lifestyle class (10.8% vs. 0.8%, *p* < 0.001). Compared to those with a lower-risk lifestyle, moderate-risk lifestyle participants more frequently reported being insufficiently active (40.1% vs. 14.9%, *p* < 0.001) or having a BMI ≥ 30 kg/m^2^ (39.0% vs. 26.3%, *p* = 0.003).

### 3.5. High-Risk Lifestyle

Participants classified in the high-risk lifestyle class did not adhere to most of the NCCN guidelines for multiple health behaviors. Compared to the lower-risk lifestyle class, the high-risk lifestyle class contained a higher proportion of participants who had a BMI ≥ 30 kg/m^2^ (43.6 vs. 26.3%, *p* = 0.016) and who were either insufficiently active (36.4% vs. 21.4%, *p* = 0.027) or moderately active (30.9% vs. 14.9%, *p* = 0.014) (Table 2 and Table 3). Participants in the high-risk lifestyle class had a significantly higher proportion of current smokers compared to both the moderate-risk (23.6% vs. 10.8%, *p* = 0.007) and lower-risk (23.6% vs. 0.8%, *p* < 0.001) lifestyle classes. Conversely, the percentage of participants in the high-risk lifestyle class who did not consume alcohol was approximately double that of the moderate-risk (60.0% vs. 37.2%, *p* = 0.002) and lower-risk (60.0% vs. 29.8%, *p* < 0.001) lifestyle classes. Although a larger proportion of the high-risk participants were non-adherent to the NCCN guidelines for a healthy diet intake compared to moderate-risk participants (18.2% vs. 1.2%, *p* < 0.001), they reported eating more fruits (mean ± *SD*: 3.3 ± 1.5 vs. 3.0 ± 1.1, *p* = 0.046), vegetables (mean ± *SD*: 3.7 ± 1.4 vs. 3.5 ± 1.0, *p* = 0.037), and whole grains (mean ± *SD*: 3.6 ± 1.5 vs. 2.8 ± 1.1, *p* < 0.001), on average, than moderate-risk lifestyle participants. A significantly higher proportion of participants with a high-risk lifestyle reported visiting their physician more than two years ago compared to the lower-risk lifestyle class (12.7% vs. 4.2%, *p* = 0.039). However, no significant differences (*p* > 0.05) were observed between high- and moderate-risk participants with respect to the last physician visit (7.6% vs. 12.7%). Those who were non-adherent to NCCN guidelines for BMI (74.7% vs. 81.8%), physical activity (61.3% vs. 67.3%), and sun safety (35.8% vs. 36.4%).

### 3.6. Sociodemographic and Health History Characteristics of Latent Classes

#### High-Risk versus Lower-Risk Lifestyle Class Comparison

**Figure 1:**
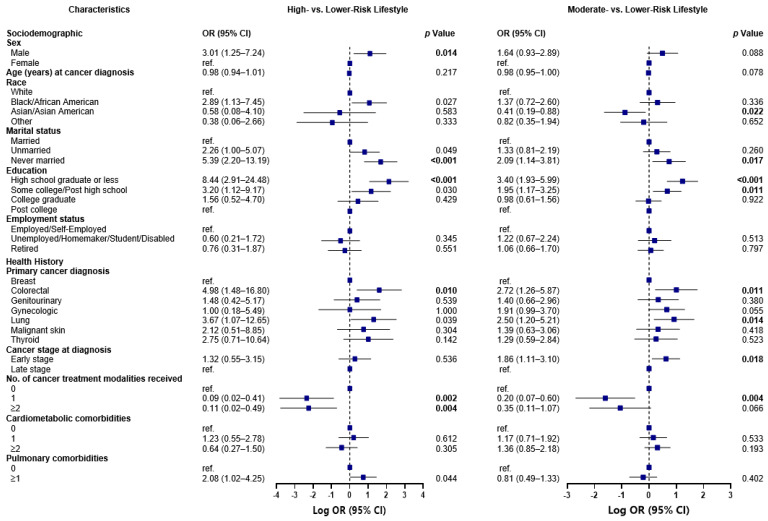
Odds of membership to the high-risk and moderate-risk lifestyle classes compared to the lower-risk lifestyle class, by sociodemographic and health history characteristics, *N* = 661. Associations between sociodemographic and health history factors and health behavior classes were examined using latent class regression, by running a generalized logit model with a multinomial distribution, and robust error variances. Associations were reported as odds ratios. Each plot illustrates the log odds of belonging to the high-risk lifestyle class and the moderate-risk lifestyle class, when compared to the log odds of belonging to the lower-risk lifestyle class. The Benjamini–Hochberg (BH) approach was used to correct for multiple comparisons, and each significant *p*-value following correction at a significance level of 5% is bolded.

Several sociodemographic and health history factors were significantly associated with class membership: the odds of living a high-risk versus lower-risk lifestyle was 3.01 times greater for males than females (OR 3.01, 95% CI 1.25–7.24; *p* = 0.014), 5.39 times greater for never married than married participants (OR 5.39, 95% CI 2.20–13.19; *p* < 0.001), and 8.44 times greater for participants with a high school diploma or less as opposed to a post college education (OR 8.44, 95% CI 2.91–24.48; *p* < 0.001) (Figure 1). Although not statistically significant following BH correction for multiple comparisons, the odds of belonging to the high-risk lifestyle class was also almost 3-fold greater for Blacks/African Americans than Whites (OR 2.89, 95% CI 1.13–7.45; *p* = 0.027), 2.26 times greater for unmarried compared to married participants (OR 2.26, 95% CI 1.00–5.07; *p* = 0.049), more than 3-fold greater for those with some college/post-high school education relative to a post-college education (OR 3.20, 95% CI 1.12–9.17; *p* = 0.030), and twice as high for participants with ≥1 vs. 0 pulmonary comorbidities (OR 2.08, 95% CI 1.02–4.25; *p* = 0.044).

Furthermore, in comparison to participants with a breast cancer diagnosis, those who were diagnosed with colorectal cancer (OR 4.98, 95% CI 1.48–16.80; *p* = 0.010) and lung cancer (OR 3.67, 95% CI 1.07–12.65; *p* = 0.039) had increased odds of belonging to the high-risk versus lower-risk lifestyle class, albeit the latter association was not statistically significant after BH adjustment. What was significant, however, was that the odds of living a high-risk lifestyle was 91% lower among those who received any form of cancer treatment, including surgery, chemotherapy, or radiation therapy (OR 0.09, 95% CI 0.02–0.41; *p* = 0.002), and 89% lower among those who received ≥2 vs. 0 of the aforementioned cancer treatment modalities (OR 0.11, 95% CI 0.02–0.49; *p* = 0.004).

### 3.7. Moderate-Risk versus Lower-Risk Lifestyle Class Comparison

There were also several notable characteristics that distinguished participants with a moderate-risk lifestyle from those with a lower-risk lifestyle (Figure 1). Compared to those in the lower-risk lifestyle, the odds of participants living a moderate-risk lifestyle was 59% lower for Asians/Asian Americans than Whites (OR 0.41, 95% CI 0.19–0.88; *p* = 0.022) and twice as high for never married than married participants (OR 2.09, 95% CI 1.14–3.81; *p* = 0.017). Additionally, the odds of living a moderate-risk lifestyle were more than 3-fold greater for participants with a high school diploma or less (OR 3.40, 95% CI 1.93–5.99; *p* < 0.001) and almost twice as high for participants with some college/post-high school education (OR 1.95, 95% CI 1.17–3.25; *p* = 0.011) relative to a post college education. In comparison to participants who received a breast cancer diagnosis, those who received colorectal and lung cancer diagnoses had more than 2-fold higher (OR 2.72, 95% CI 1.26–5.87; *p* = 0.011) and two and a half times (OR 2.50, 95% CI 1.20–5.21; *p* = 0.014) greater odds of belonging to the moderate-risk versus lower-risk lifestyle class. The odds of living a moderate-risk lifestyle were almost 2-fold greater for participants with early versus late-stage cancer at diagnosis (OR 1.86, 95% CI 1.11–3.10; *p* = 0.018) and 80% lower among those who received any form of cancer treatment (OR 0.20, 95% CI 0.07–0.60; *p* = 0.004).

### 3.8. High-Risk vs. Moderate-Risk Lifestyle Class Comparison

**Figure 2:**
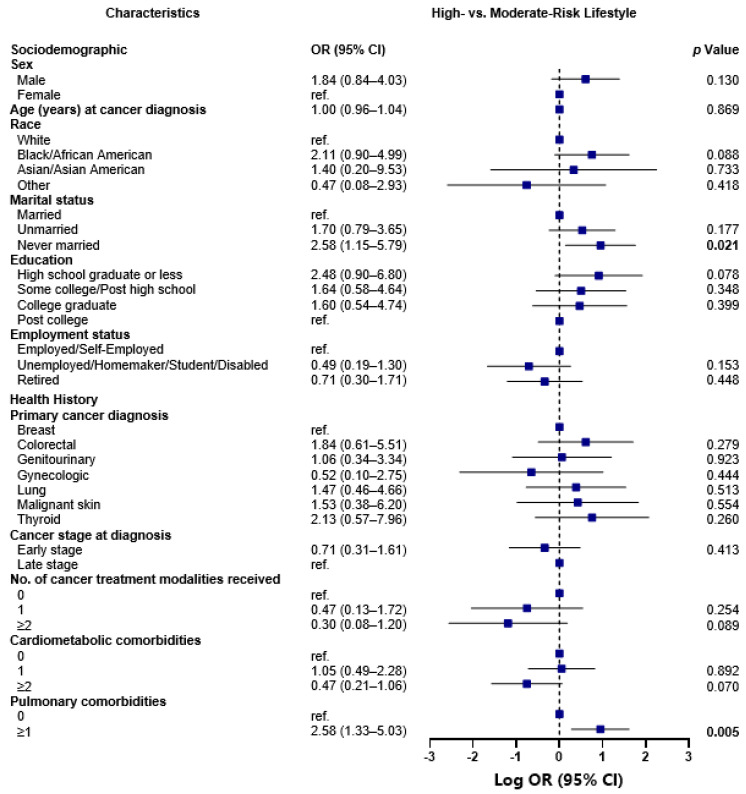
Odds of membership to the high-risk versus moderate-risk lifestyle class, by sociodemographic and health history characteristics, *N* = 661. Associations between sociodemographic and health history factors and health behavior classes were examined using latent class regression, by running a generalized logit model with a multinomial distribution, and robust error variances. Associations were reported as odds ratios. The plot illustrates the log odds of belonging to the high-risk lifestyle class when compared to the log odds of belonging to the moderate-risk lifestyle class. The Benjamini–Hochberg (BH) approach was used to correct for multiple comparisons, and each significant p-value following correction at a significance level of 5% is bolded.

The high-risk and moderate-risk lifestyle classes were more alike with respect to the measured sociodemographic and health history characteristics than either one was in comparison to the lower-risk lifestyle class (Figure 2). The only significant differences to note were as follows: the odds of living a high-risk versus moderate-risk lifestyle were more than 2-fold greater for never married than married participants (OR 2.58, 95% CI 1.15–5.79; *p* = 0.021) and for participants with ≥1 vs. 0 pulmonary comorbidities (OR 2.58, 95% CI 1.33–5.03; *p* = 0.005).

## 4. Discussion

This cross-sectional study identified three classes of multiple health behavior adherence among cancer survivors. Almost 40% of the participants fit in the lower-risk lifestyle class characterized by adherence to all health behaviors except for alcohol consumption. Approximately half of the surveyed participants (52%) fit in the moderate risk lifestyle class characterized by a healthy dietary intake that was low in sugar, red meat, processed meat and fast foods, never smoking, and being insufficiently active. Participants (8.3%) who fit the high-risk lifestyle class characterized by a BMI ≥30 kg/m^2^ were insufficiently or moderately active, current smokers, and consumed alcohol. Relative to both low- and high-risk lifestyle classes, those in the moderate-risk lifestyle class were more likely to self-identify as a race other than Asian/Asian American, were married, earned a high school diploma or less, were diagnosed with colorectal cancer or lung cancer, and were diagnosed with later stage cancer. Similarly, relative to both low- and moderate-risk lifestyle classes, those in the high-risk lifestyle class were more likely to be male, never married, have a high school diploma or less, diagnosed with colorectal or lung cancers, and have pulmonary comorbidities. Although not significant after BH correction, Black/African American survivors were more likely to belong to the high-risk health behavior lifestyle class. Since the high-risk lifestyle class had lower membership, the membership characteristics are discussed with the moderate-risk lifestyle class.

Most participants surveyed belonged to the moderate-risk lifestyle class and adhered to some of the NCCN guidelines for multiple health behaviors, which contrasts with the previous literature [15,32,33]. A small study (*N* = 66) of cancer survivors from a survivorship care clinic found that only 7.6% were adherent to all six NCCN multiple health behavior guidelines [32]. In comparison, our sample in the moderate-risk lifestyle class, which was the largest class, adhered to five of seven health behaviors. Interestingly, participants in the moderate-risk lifestyle class reported consuming a relatively healthy diet that was low in sugar, red and processed meats, and fast foods compared to those in the high-risk lifestyle class. However, participants in the moderate-risk lifestyle class also had low adherence to recommendations for fruit, vegetable, and whole grain consumption. Consuming a diet that is rich in plants and low in processed foods is important for cancer survivorship and promotes maintaining a healthy BMI, reducing cardiovascular risks, and improvement in cancer-related biomarkers [9,34,35,36]. Moreover, participants in this class were non-adherent to physical activity and BMI guidelines. While the reported diets may be low in processed foods, participants in the moderate-risk lifestyle class are not consuming enough nutrient dense foods to meet guidelines. Which combined with the non-adherence to physical activity guidelines, may explain the higher mean BMI of participants in this class. Future research is encouraged to develop intervention strategies to promote healthy dietary patterns and physical activity through behavior change, specifically focusing on facilitating weight maintenance concordant with NCCN guidelines and to determine the impacts on long-term prognosis [13].

There were several sociodemographic and SES characteristics that were associated with membership in the moderate- and high-risk lifestyle classes, such as sex, marital status, education, and race. Among the general population without a cancer diagnosis, it is well-established that males are more likely to report riskier health behaviors, such as smoking and drinking, compared to females [37]. However, sex differences in multiple health behavior change between males and females has yet to be explored thoroughly among cancer survivors [15,32]. Interpersonal relationships through marital status may also buffer against non-adherence through provision of social support for health behaviors [13]. The current findings are consistent with observations among cancer survivors where lower education attainment is significantly associated with non-adherence to multiple health behaviors [36]. Additionally, consistent with previous research [24,38,39], race and ethnicity were associated with increased likelihood for multiple health behavior non-adherence. In our sample, Asians/Asian Americans were more likely to be in the moderate-risk lifestyle class and there were trends for Black/African American individuals being in the high-risk lifestyle class. Racial and ethnic differences were not observed for membership in the lower-risk lifestyle class. In a previous study, Asian or Asian American breast cancer survivors reported having a trustworthy relationship with their medical provider and family members, which was significantly associated with intentions to engage in healthy behaviors [40]. Moreover, such a relationship with medical providers is often absent among Black cancer survivors [38]. It is plausible that the interpersonal relationships with medical providers may buffer against increased non-adherence to NCCN multiple health behavior guidelines among Asian or Asian American cancer survivors in the current study [32]. Future longitudinal studies are needed to examine associations and predictors of adherence to multiple health behaviors with a larger sample that is racially and socioeconomically diverse to determine which significant variables are maintained to identify at-risk subpopulations. Further, racial and socioeconomic variables should also be considered when developing multiple health behavior change interventions.

Key cancer- and comorbidity-related factors of membership in either moderate- or high-risk lifestyle classes included diagnosis with later stage colorectal cancer or lung cancer and having a history of pulmonary comorbidities. Diagnoses of both cancer and additional comorbidities increases the burden of poorer cancer outcomes and decreases health-related quality of life for many survivors [36]. Engaging in multiple health behaviors is particularly advantageous for cancer survivors through potential to mitigate cumulative risks for both cancer and overall health [13]. The current findings are reflective of previous observations highlighting the complex interaction between a cancer diagnosis, diagnosis with cardiometabolic comorbidities, and multiple health behavior adherence [15], which should be considered in-clinic when health care providers discuss health behaviors recommendations [41]. Future research needs to prioritize health behavior discussions between health care providers and cancer survivors with comorbidities and experiencing health disparities with respect to sociodemographic and SES characteristics. To facilitate NCCN guidelines adherence among cancer survivors, future research is encouraged to develop and test intervention strategies that promote effective guideline concordant recommendations from health care providers.

This study has limitations that need to be considered, including very few cancer survivors with Hispanic ethnicity being sampled, which is partially attributed with the inability to provide Spanish language translation. However, the results related to race are compelling given the smaller proportion of racially and ethnically diverse representation and highlight important racial disparities among the sample. Relying on self-report measures may have led to recall bias. Further, selection bias may have been induced by those who are both interested in health behaviors and had the time to complete a paper-and-pencil survey, as reflected by the 28.6% response rate. We used a cross-sectional study design and causal inferences cannot be made. The study could be strengthened by linking responders’ questionnaire data to electronic medical record data. Strengths include a population-based study sample that included socioeconomic variation which is reflective of the geographical region. The study relied on validated measures to assess the outcomes, which is an indicator of rigor. Finally, most of the recommended health behaviors from the NCCN guidelines, except for sleep, were examined in this study, which is comprehensive.

## 5. Conclusions

A dearth of evidence exists which identifies sociodemographic, cancer-, and comorbidity-related characteristics of multiple health behavior adherence among cancer survivors. Our latent class analysis identified three classes of adherence to NCCN guidelines for multiple health behaviors among a sample of cancer survivors in New Jersey. Participants in the moderate- and high-risk lifestyle classes were more likely to be male with a high school diploma or less, unmarried, or never married, self-identified as Asian/Asian American, diagnosed with colorectal cancer or lung cancers at a later stage, and have a history of pulmonary comorbidities. The findings highlight the need to develop interventions and in-clinic strategies which facilitate multiple health behavior adherence among this at-risk group.

## Figures and Tables

**Table 1:** Descriptive statistics of New Jersey cancer survivor cohort members, *N* = 661.

Characteristics	Overall *N* (%)	Missing *N* (%)
Sociodemographic		
**Sex**		0 (0.0)
Male	273 (41.3)	
Female	388 (58.7)	
**Age (years) at cancer diagnosis, *mean ± SD***	60.8 ± 10.5	0 (0.0)
**Race**		0 (0.0)
White	518 (78.4)	
Black/African American	75 (11.3)	
Asian/Asian American	39 (5.9)	
Other **^a^**	29 (4.4)	
**Hispanic ethnicity**		0 (0.0)
Yes	39 (5.9)	
No	622 (94.1)	
**US-born**		2 (0.3)
Yes	569 (86.1)	
No	88 (13.3)	
Prefer not to answer	2 (0.3)	
**Marital status**		2 (0.3)
Married	452 (68.4)	
Unmarried	119 (18.0)	
Never married	82 (12.4)	
Don’t know/Prefer not to answer	6 (0.9)	
**Education ^b^**		4 (0.6)
High school graduate or less	149 (22.5)	
Some college/Post high school	152 (23.0)	
College graduate	201 (30.4)	
Post college	150 (22.7)	
Other-unspecified/Don’t know/Prefer not to answer	5 (0.8)	
**Employment status**		12 (1.8)
Employed/Self-Employed	284 (43.0)	
Unemployed/Homemaker/Student/Disabled	84 (12.7)	
Retired	277 (41.9)	
Other-unspecified/Don’t know/Prefer not to answer	4 (0.6)	
**Annual household income**		11 (1.7)
<$50,000	161 (24.4)	
$50,000–$89,999	151 (22.8)	
≥$90,000	211 (31.9)	
Don’t know	127 (19.2)	
**Health insurance status at cancer diagnosis**		0 (0.0)
Uninsured	8 (1.2)	
Private	312 (47.2)	
Public	277 (41.9)	
Insured, not otherwise specified	40 (6.1)	
Don’t know	24 (3.6)	
**Health History**		
**Primary cancer diagnosis**		0 (0.0)
Breast	169 (25.6)	
Colorectal	66 (10.0)	
Genitourinary **^c^**	183 (27.7)	
Gynecologic **^d^**	67 (10.1)	
Lung	66 (10.0)	
Malignant skin	66 (10.0)	
Thyroid	44 (6.7)	
**Cancer stage at diagnosis ^e^**		0 (0.0)
Early stage	515 (77.9)	
Late stage	137 (20.7)	
Unstaged	9 (1.4)	
**Time (years) since cancer diagnosis, *mean ± SD***	3.2 ± 0.7	0 (0.0)
**Cancer treatment type**		
Surgery		15 (2.3)
Yes	545 (82.5)	
No	100 (15.1)	
Do not recall	1 (0.2)	
Chemotherapy		62 (9.4)
Yes	179 (27.1)	
No	418 (63.2)	
Do not recall	2 (0.3)	
Radiotherapy		43 (6.5)
Yes	258 (39.0)	
No	359 (54.3)	
Do not recall	1 (0.2)	
**No. of cancer treatment modalities received**		53 (8.0)
0	27 (4.1)	
1	307 (46.4)	
≥2	274 (41.5)	
**Cardiometabolic comorbidities ^f^**		14 (2.1)
0	188 (28.4)	
1	174 (26.3)	
≥2	285 (43.1)	
**Pulmonary comorbidities ^g^**		12 (1.8)
0	529 (80.0)	
≥1	120 (18.2)	
**Other comorbidities ^h^**		16 (2.4)
0	446 (67.5)	
≥1	199 (30.1)	

^a^ Other race includes those self-identifying as other (*N* = 14) or multiracial (*N* = 15). ^b^ High school graduate or less includes those who graduated (*N* = 125) and those who did not (*N* = 23). Post college includes individuals who completed a graduate degree, professional degree, or other certifications/credits following a college degree. ^c^ Genitourinary cancers include bladder (*N* = 63) and prostate (*N* = 120). ^d^ Gynecologic cancers include vulvar (*N* = 6), endocervical (*N* = 2), cervical (*N* = 5), endometrial (*N* = 44), myometrium (*N* = 1), and ovarian (*N* = 9). ^e^ Cancer stage at diagnosis is divided into early stage, including in situ (*N* = 48) and localized (*N* = 467) cases, as well as late stage, including regional (*N* = 130) and distant (*N* = 7) cases. ^f^ Cardiometabolic comorbidities include one or more of the following: history of diabetes (*N* = 121); hypertension (*N* = 342); hypercholesterolemia (*N* = 295); heart disease, angina, or heart attack (*N* = 86); congestive heart failure (*N* = 18); myocardial infarction (*N* = 11); kidney disease (*N* = 40); and/or liver disease (*N* = 10). ^g^ Pulmonary comorbidities include one or more of the following: history of emphysema/chronic obstructive pulmonary disease (COPD) (*N* = 58); and/or asthma (*N* = 82). ^h^ Other comorbidities include one or more of the following: history of depression (*N* = 103); anxiety disorder (*N* = 95); schizophrenia (*N* = 1); bipolar disorder (*N* = 10); post-traumatic stress disorder (PTSD) (*N* = 21); peripheral vascular disease (*N* = 14); cerebrovascular disease (*N* = 4); dementia (*N* = 4); connective tissue disease (*N* = 5); leukemia (*N* = 2); malignant lymphoma (*N* = 6); hematological or solid tumor (*N* = 60); and/or acquired immunodeficiency syndrome (AIDS) (*N* = 3).

**Table 2:** Health behavior patterns among New Jersey cancer survivors, overall and by latent class membership.

BMI and Health Behaviors	Overall *N* (%)	Health Behavior Class ^†^		
		High-Risk Lifestyle A (%)	Moderate-Risk Lifestyle B (%)	Lower-Risk Lifestyle C (%)
**Latent Class Membership**	661 (100.0)	55 (8.3)	344 (52.0)	262 (39.6)
**BMI (kg/m^2^) ^a^**				
<25.0	194 (29.3)	11 (20.0)	90 (26.2)	93 (35.5) ^A,B^
25.0–29.9	240 (36.3)	20 (36.4)	120 (34.9)	100 (38.2)
≥30.0	227 (34.3)	24 (43.6) ^C^	134 (39.0) ^C^	69 (26.3)
**Physical activity**				
Insufficiently active/sedentary (LSI < 14)	214 (32.4)	20 (36.4) ^C^	138 (40.1) ^C^	56 (21.4)
Moderately active (LSI = 14–23)	129 (19.5)	17 (30.9) ^C^	73 (21.2)	39 (14.9)
Active (LSI ≥ 24)	318 (48.1)	18 (32.7)	133 (38.7)	167 (63.7) ^A,B^
**Smoking status**				
Never smoker	340 (51.4)	17 (30.9)	168 (48.8) ^A^	155 (59.2) ^A,B^
Former smoker	269 (40.7)	25 (45.5)	139 (40.4)	105 (40.1)
Current smoker	52 (7.9)	13 (23.6) ^B,C^	37 (10.8) ^C^	2 (0.8)
**Alcohol consumption**				
0 drinks/day	239 (36.2)	33 (60.0) ^B,C^	128 (37.2)	78 (29.8)
0 < drinks/day ≤ 1	197 (29.8)	11 (20.0)	101 (29.4)	85 (32.4)
1 < drinks/day ≤ 2	150 (22.7)	8 (14.5)	72 (20.9)	70 (26.7)
>2 drinks/day	75 (11.3)	3 (5.5)	43 (12.5)	29 (11.1)
**Healthy diet intake ^b^**				
More fruit, *mean ± SD*	3.9 ± 1.5	3.3 ± 1.5 ^B^	3.0 ± 1.1	5.1 ± 0.9 ^A,B^
More vegetables, *mean ± SD*	4.3 ± 1.3	3.7 ± 1.4 ^B^	3.5 ± 1.0	5.4 ± 0.6 ^A,B^
More whole grains, *mean ± SD*	3.5 ± 1.4	3.6 ± 1.5 ^B^	2.8 ± 1.1	4.2 ± 1.4 ^A,B^
Less sugar, *mean ± SD*	5.4 ± 1.1	2.2 ± 0.8	5.5 ± 0.7 ^A^	5.8 ± 0.5 ^A,B^
Less red meat, *mean ± SD*	4.4 ± 1.0	3.7 ± 1.3	4.3 ± 0.8 ^A^	4.6 ± 1.0 ^A,B^
Less processed meat, *mean ± SD*	4.8 ± 0.9	4.1 ± 1.2	4.6 ± 0.9 ^A^	5.1 ± 0.9 ^A,B^
Less fast foods, *mean ± SD*	5.0 ± 0.8	4.5 ± 0.9	4.9 ± 0.8 ^A^	5.3 ± 0.6 ^A,B^
**Sun safety ^c^**				
Wearing sunscreen, *mean ± SD*	3.0 ± 1.4	2.6 ± 1.5	2.8 ± 1.4	3.4 ± 1.3 ^A,B^
Wearing shirt with sleeves covering shoulders, *mean ± SD*	3.8 ± 1.2	3.8 ± 1.4	3.8 ± 1.2	3.8 ± 1.2
Wearing a hat, *mean ± SD*	3.0 ± 1.3	3.2 ± 1.3	2.8 ± 1.3	3.2 ± 1.3 ^B^
Staying in the shade or under an umbrella, *mean ± SD*	3.4 ± 1.0	3.2 ± 1.2	3.4 ± 1.0	3.4 ± 1.0
Wearing sunglasses, *mean ± SD*	3.9 ± 1.3	3.7 ± 1.4	3.7 ± 1.4	4.2 ± 1.1 ^A,B^
**Last physician visit**				
<4 weeks ago	150 (22.7)	16 (29.1)	75 (21.8)	59 (22.5)
1–3 months ago	204 (30.9)	17 (30.9)	101 (29.4)	86 (32.8)
4–6 months ago	176 (26.6)	11 (20.0)	93 (27.0)	72 (27.5)
7–12 months ago	87 (13.2)	4 (7.3)	49 (14.2)	34 (13.0)
>2 years ago	44 (6.7)	7 (12.7) ^C^	26 (7.6)	11 (4.2)

Abbreviations: BMI, Body Mass Index; LSI, Leisure Score Index. ^†^ Pairwise comparisons of proportions across health behavior classes were evaluated using two-sided Chi-Squared tests (assuming equal variances), whereas pairwise comparisons of means across health behavior classes were assessed using Analysis of Variance (ANOVA). The Benjamini–Hochberg procedure corrected for multiple comparisons using the 5% level of significance. For each Benjamini–Hochberg-corrected significant pair, the health behavior class with the larger proportion/mean was marked with a superscript denoting the class with the smaller proportion/mean that it was compared to. The superscripts representing each latent class of health behavior are A = high-risk lifestyle, B = moderate-risk lifestyle, and C = lower-risk lifestyle. ^a^ For BMI <25.0 kg/m^2^, the following groups were combined: <18.5 kg/m^2^ (underweight, *N* = 5) and 18.5–25.0 kg/m^2^ (normal weight, *N* = 189). BMI 25.0–29.9 kg/m^2^ and ≥30.0 kg/m^2^ comprised of overweight and obese individuals, respectively. ^b^ A 7-item measure was used to assess food frequency over the past week, where responses included 1 = “never”, 2 = “once/week”, 3 = “2–4 times/week”, 4 = “5–6 times/week”, 5 = “once/day”, and 6 = “≥2 times/day”. Sugar-sweetened beverages, red meat, processed meat, and fast foods items were reverse coded. Items with a higher mean score indicate closer alignment with nutritional recommendations from the World Cancer Research Fund/American Institute of Cancer Research. ^c^ Five items from the Health Information National Trends Survey (HINTS) were used to assess sun safety. Responses were collected using a 5-point Likert scale, where 1 = “never”, 2 = “rarely”, 3 = “sometimes”, 4 = “often”, and 5 = “always.”.

**Table 3:** NCCN health behavior guidelines and adherence patterns among New Jersey cancer survivors, overall and by latent class membership.

NCCN Health Behavior Guidelines		Adherence Criteria	Overall *N* (%)	Health Behavior Class ^†^		
				High-Risk Lifestyle A (%)	Moderate-Risk Lifestyle B (%)	Lower-Risk Lifestyle C (%)
**Latent Class Membership**			**661 (100.0)**	55 (8.3)	344 (52.0)	262 (39.6)
**BMI**	Achieve and maintain a healthy body weight throughout life.	**Adherent:** Healthy weight (18.5–24.9 kg/m^2^)	189 (28.6)	10 (18.2%)	87 (25.3%)	92 (35.1%) ^A,B^
		**Non-Adherent:** Underweight (<18.5 kg/m^2^), Overweight (25.0–29.9 kg/m^2^), or Obese (≥30.0 kg/m^2^)	472 (71.4)	45 (81.8%) ^C^	257 (74.7%) ^C^	170 (64.9%)
**Physical activity**	Regularly exercise. Stay active. Aim for 150 min of moderate or 75 min of vigorous activity per week, spread out over the course of the week. Resistance train 2–3 times a week and stretch major muscles on at least two days of higher-intensity activity.	**Adherent:** Active (LSI≥24)	318 (48.1)	18 (32.7%)	133 (38.7%)	167 (63.7%) ^A,B^
		**Non-Adherent:** Insufficiently/Moderately active (LSI<24)	343 (51.9)	37 (67.3%) ^C^	211 (61.3%) ^C^	95 (36.3%)
**Smoking status**	Don’t smoke, chew, or sniff tobacco products. Attempt tobacco cessation if currently smoking or using smokeless tobacco.	**Adherent:** Not currently smoking	609 (92.1)	42 (76.4%)	307 (89.2%) ^A^	260 (99.2%) ^A,B^
		**Non-Adherent:** Currently smoking	52 (7.9)	13 (23.6%) ^B,C^	37 (10.8%) ^C^	2 (0.8%)
**Alcohol consumption**	Limit or abstain from alcohol intake; limit intake to one drink/day for a woman and 2 drinks/day for a man.	**Adherent:** 1/day for females; 2/day for males	504 (76.2)	46 (83.6%)	268 (77.9%)	190 (72.5%)
		**Non-Adherent:** >1/day for females; >2/day for males	157 (23.8)	9 (16.4%)	76 (22.1%)	72 (27.5%)
**Healthy diet intake**	Aim for at least half of your diet to be plant-based food. Plant-based food should be mostly vegetables, fruits, and whole grains. Half or less of your diet can be animal-based food—fish and poultry are healthy choices. Limit intake of red and processed meats. Avoid processed food. Drinks with lots of added sugars or fats should be consumed sparingly.	**Adherent:** Mean score >3	647 (97.9)	45 (81.8%)	340 (98.8%)^A^	262 (100.0%) *
		**Non-Adherent:** Mean score ≤3	14 (2.1)	10 (18.2%) ^B^	4 (1.2%)	0 (0.0%) *
**Sun safety**	Practice sun safety. Use water-resistant, UVA/UVB-protecting sunscreen with at least 30 SPF. Apply generously and reapply every 2 h or after swimming/excessive sweating. If possible, consider physical barriers (i.e., hats, shirts with sleeves, avoiding direct sun during peak hours).	**Adherent:** Mean score >3	465 (70.3)	35 (63.6%)	221 (64.2%)	209 (79.8%) ^A,B^
		**Non-Adherent:** Mean score ≤3	196 (29.7)	20 (36.4%)^C^	123 (35.8%)^C^	53 (20.2%)
**Last physician visit**	Follow up with primary care physician regularly.	**Adherent:** <4 weeks ago to 12 months ago	617 (93.3)	48 (87.3%)	318 (92.4%)	251 (95.8%)^A^
		**Non-Adherent:** >2 years ago	44 (6.7)	7 (12.7%)^C^	26 (7.6%)	11 (4.2%)

Abbreviations: BMI, Body Mass Index; LSI, Leisure Score Index. ^†^ Pairwise comparisons of proportions across health behavior classes were evaluated using two-sided Chi-Squared tests (assuming equal variances), with the exception of cells where the proportion was either zero or one (marked by asterisks). The Benjamini–Hochberg procedure corrected for multiple comparisons using the 5% level of significance. For each Benjamini–Hochberg-corrected significant pair, the health behavior class with the larger proportion/mean was marked with a superscript denoting the class with the smaller proportion/mean that it was compared to. The superscripts representing each latent class of health behavior are A = high-risk lifestyle, B = moderate-risk lifestyle, and C = lower-risk lifestyle.

## Data Availability

The data presented in this study are available on request from the corresponding author. The data are not publicly available due to privacy or ethical restrictions.

## Supplementary Materials

The following supporting information can be downloaded at: https://www.mdpi.com/article/10.3390/nu15102354/s1, Table S1: Comparison of characteristics among New Jersey cancer survivor cohort members, between non-responders and responders, and those included versus excluded from the analytic sample, *N* = 2692; Table S2: Evaluating Class Solutions, *N* = 661; Table S3: Latent class analysis item response probabilities and means for each health behavior class, *N* = 661.
