# Supplementary material for: Sociodemographic and Health Correlates of Multiple Health Behavior Adherence among Cancer Survivors: A Latent Class Analysis
Source: Nutrients. 2023 May 17;15(10):2354. doi: 10.3390/nu15102354 (PMC10223681; doi:10.3390/nu15102354)
Supplement: Supplementary file 1 [file nutrients-15-02354-s001.zip › nutrients-2324785-supplementary.pdf]

SUPPLEMENTARY TABLES

Supplementary Table S1. Comparison of characteristics among New Jersey cancer survivor cohort members, between non-responders and responders, and those included versus excluded from the analytic sample, N=2,692

CHARACTERISTICS	Non-Responders N (%)[†]	Responders		*P*-value[††]	
		Included N (%)[†]	Excluded N (%)[†]	Non-Responders vs. Included Responders	Excluded vs. Included Responders
**TOTAL**	1830 (100.0)	661 (100.0)	201 (100.0)		
**SOCIODEMOGRAPHIC**					
**Sex**				0.477	0.614
Male	785 (42.9)	273 (41.3)	79 (39.3)		
Female	1045 (57.1)	388 (58.7)	122 (60.7)		
**Age (years) at cancer diagnosis**				0.054	0.298
18-24	17 (0.9)	4 (0.6)	3 (1.5)		
25-44	161 (8.8)	45 (6.8)	13 (6.5)		
45-64	953 (52.1)	324 (48.9)	109 (54.2)		
≥65	699 (38.2)	289 (43.7)	76 (37.8)		
**Race**				**<0.001**	0.131
White	1341 (73.3)	518 (78.4)	149 (74.1)		
Black/African American	330 (18.0)	75 (11.3)	34 (16.9)		
Asian/Asian American	158 (8.6)	39 (5.9)	13 (6.5)		
Other[a]	1 (0.1)	29 (4.4)	5 (2.5)		
**Hispanic ethnicity**				0.383	0.971
Yes	126 (6.9)	39 (5.9)	12 (6.0)		
No	1704 (93.1)	622 (94.1)	189 (94.0)		
**US-born**	NA			NA	0.341
Yes		569 (86.1)	165 (82.1)		
No		88 (13.3)	30 (14.9)		
Prefer not to answer		2 (0.3)	2 (1.0)		
**Marital status**	NA			NA	0.585
Married		452 (68.4)	125 (62.2)		
Unmarried		119 (18.0)	43 (21.4)		
Never married		82 (12.4)	27 (13.4)		
Don't know/Prefer not to answer		6 (0.9)	2 (1.0)		
**Education[b]**	NA			NA	0.398
High school graduate or less		149 (22.5)	45 (22.4)		
Some college/Post high school		152 (23.0)	53 (26.4)		
College graduate		201 (30.4)	59 (29.4)		
Post college		150 (22.7)	38 (18.9)		
Other-unspecified/Don't know/Prefer not to answer		5 (0.8)	4 (2.0)		
**Employment status**	NA			NA	0.563
Employed/Self-Employed		284 (43.0)	89 (44.3)		
Unemployed/Homemaker/Student/Disabled		84 (12.7)	31 (15.4)		
Retired		277 (41.9)	75 (37.3)		
Other-unspecified/Don't know/Prefer not to answer		4 (0.6)	2 (1.0)		
**Household income**	NA			NA	**0.027**
<$50,000		161 (24.4)	43 (21.4)		
$50,000 - $89,999		151 (22.8)	40 (19.9)		
≥$90,000		211 (31.9)	54 (26.9)		
Don't know		127 (19.2)	58 (28.9)		
**Health insurance status at cancer diagnosis**	NA			NA	0.508
Uninsured		8 (1.2)	4 (2.0)		
Private		312 (47.2)	92 (45.8)		
Public		277 (41.9)	74 (36.8)		
Insured, not otherwise specified		40 (6.1)	17 (8.5)		
Don't know		24 (3.6)	9 (4.5)		
**HEALTH HISTORY**					
**Primary cancer diagnosis**				**0.004**	0.200
Breast	350 (19.1)	169 (25.6)	46 (22.9)		
Colorectal	224 (12.2)	66 (10.0)	33 (16.4)		
Genitourinary[c]	505 (27.6)	183 (27.7)	51 (25.4)		
Gynecologic[d]	239 (13.1)	67 (10.1)	21 (10.4)		
Lung	166 (9.1)	66 (10.0)	20 (10.0)		
Malignant skin	196 (10.7)	66 (10.0)	12 (6.0)		
Thyroid	150 (8.2)	44 (6.7)	18 (9.0)		
**Cancer stage at diagnosis[e]**				0.499	**0.037**
Early stage	1385 (75.7)	515 (77.9)	140 (69.7)		
Late stage	420 (23.0)	137 (20.7)	59 (29.4)		
Unstaged	25 (1.4)	9 (1.4)	2 (1.0)		
**Time (years) since cancer diagnosis, *mean±SD***	NA	3.2±0.7	3.2±0.8	NA	0.565
**Cancer treatment type**					
Surgery	NA			NA	0.732
Yes		545 (82.5)	168 (83.6)		

No		100 (15.1)	27 (13.4)		
Do not recall		1 (0.2)	0 (0.0)		
Chemotherapy	NA			NA	0.061
Yes		179 (27.1)	71 (35.3)		
No		418 (63.2)	110 (54.7)		
Do not recall		2 (0.3)	1 (0.5)		
Radiotherapy	NA			NA	0.831
Yes		258 (39.0)	81 (40.3)		
No		359 (54.3)	108 (53.7)		
Do not recall		1 (0.2)	0 (0.0)		
**No. of cancer treatment modalities received**	NA			NA	0.301
0		27 (4.1)	5 (2.5)		
1		307 (46.4)	87 (43.3)		
≥2		274 (41.5)	94 (46.8)		
**Cardiometabolic comorbidities**[f]	NA			NA	0.565
0		188 (28.4)	59 (29.4)		
1		174 (26.3)	45 (22.4)		
≥2		285 (43.1)	91 (45.3)		
**Pulmonary comorbidities**[g]	NA			NA	0.171
0		529 (80.0)	144 (71.6)		
≥1		120 (18.2)	43 (21.4)		
**Other comorbidities**[h]	NA			NA	0.782
0		446 (67.5)	128 (63.7)		
≥1		199 (30.1)	60 (29.9)		

Abbreviations: NA, Not Available

[†] Column frequencies and percents for observations may not add up to the total N or 100% if there are missing values for any given variable.

[††] Comparisons for all categorical variables used Chi-Squared tests and comparisons for continuous variables used Analysis of Variance (ANOVA). *P*-values <0.05 are bolded.

[a] Other race includes those self-identifying as other or multiracial.

[b] Post college includes individuals who completed a graduate degree, professional degree, or other certifications/credits following a college degree.

[c] Genitourinary cancers includes bladder and prostate.

[d] Gynecologic cancers include vulvar, endocervical, cervical, endometrial, myometrium, and ovarian.

[e] Cancer stage at diagnosis is divided into early stage, including in situ and localized cases, as well as late stage, including regional and distant cases.

[f] Cardiometabolic comorbidities includes history of diabetes, hypertension, hypercholesterolemia, heart disease, angina, heart attack, congestive heart failure, myocardial infarction, kidney disease, and/or liver disease.

[g] Pulmonary comorbidities includes history of emphysema/chronic obstructive pulmonary disease (COPD), and/or asthma.

[h] Other comorbidities includes history of depression, anxiety disorder, schizophrenia, bipolar disorder, post-traumatic stress disorder (PTSD), peripheral vascular disease. cerebrovascular disease, dementia, connective tissue disease, leukemia, malignant lymphoma, hematological or solid tumor, and/or acquired immunodeficiency syndrome (AIDS).

Supplementary Table S2. Evaluating Class Solutions, N=661

NUMBER OF CLASSES†	BIC	VLMR-LRT *P-Value*	Smallest Class Size, N (%)
2	32528.76	<0.0001	296 (44.8%)
**3**	**32243.71**	**<0.0001**	**55 (8.3%)**
4	32212.44	0.7154	54 (8.1%)
5	32227.26	0.2241	53 (8.0%)
6	32352.24	0.5083	12 (1.8%)

Abbreviations: BIC, Bayesian information criterion; VLMR LRT, Vuong-Lo-Mendell-Rubin adjusted likelihood ratio test

† Bolded text indicates model met fit criteria.

Supplementary Table S3. Latent class analysis item response probabilities and means for each health behavior class, N=661

BODY MASS INDEX (BMI) AND HEALTH BEHAVIORS	HEALTH BEHAVIOR CLASS†		
	High-risk lifestyle	Moderate-risk lifestyle	Low-risk lifestyle
**LATENT CLASS MEMBERSHIP N (%)**	55 (8.3)	344 (52.0)	262 (39.6)
**BMI (kg/m^2)[a]**			
<25.00	0.20	0.27	0.35
25.00 – 29.99	0.35	0.35	0.38
≥30.00	0.45	0.39	0.27
**Physical activity[a]**			
Insufficiently active/sedentary (LSI<14)	0.36	0.40	0.22
Moderately active (LSI=14-23)	0.31	0.21	0.15
Active (LSI≥24)	0.34	0.39	0.63
**Smoking status[a]**			
Never smoker	0.30	0.49	0.59
Former smoker	0.46	0.40	0.40
Current smoker	0.24	0.10	0.02*
**Alcohol consumption[a]**			
0 drinks/day	0.60	0.37	0.31
0 < drinks/day ≤ 1	0.20	0.30	0.32
1 < drinks/day ≤ 2	0.14	0.21	0.27
>2 drinks/day	0.07*	0.13	0.11
**Healthy diet intake[b]**			
More fruit	3.27	2.98	5.06
More vegetables	3.72	3.48	5.32
More whole grains	3.54	2.86	4.17
Less sugar	2.27	5.55	5.75
Less red meat	3.70	4.34	4.63
Less processed meat	4.11	4.65	5.04
Less fast foods	4.50	4.93	5.29
**Sun safety[b]**			
Wearing sunscreen	2.56	2.81	3.38
Wearing shirt with sleeves covering shoulders	3.79	3.78	3.84
Wearing a hat	3.18	2.83	3.15
Staying in the shade or under an umbrella	3.23	3.43	3.36
Wearing sunglasses	3.70	3.73	4.15
**Last physician visit[a]**			
<4 weeks ago	0.29	0.22	0.23
1-3 months ago	0.31	0.29	0.33
4-6 months ago	0.20	0.27	0.27
7-12 months ago	0.07	0.14	0.13
>2 years ago	0.12	0.08	0.04

† Latent class analysis identified a 3-class model as the best fit and most interpretable model for describing distinct health behavior patterns among cancer survivors in this cohort. Item response probabilities and means are reported for each latent or health behavior class. All probabilities and means were significantly different from zero except for those marked with an asterisk (*).

[a] Item response probabilities for categorical health behavior items (BMI, physical activity, smoking status, alcohol consumption, and last physician visit)

[b] Item response means for continuous health behavior items (healthy diet intake and sun safety)
